# Host-Adaptation of *Burkholderia pseudomallei* Alters Metabolism and Virulence: a Global Proteome Analysis

**DOI:** 10.1038/s41598-017-09373-0

**Published:** 2017-08-21

**Authors:** Vanitha Mariappan, Kumutha Malar Vellasamy, Jamuna Vadivelu

**Affiliations:** Department of Medical Microbiology, Faculty of Medicine, University of Malaya, 50603 Kuala Lumpur, Malaysia

## Abstract

Little is known about the evolution, adaptation and pathogenesis of *Burkholderia pseudomallei* within host during acute melioidosis infection. Melioidosis is a potential life threatening disease contracted through inhalation, ingestion, inoculation or direct entry of the organism into the blood stream via wounds or skin abrasions from contaminated soil and water. Environmental *B. pseudomallei* strain (Bp_*MARAN*_), isolated during a melioidosis outbreak in Pahang, Malaysia was injected intra-peritoneally into a mouse and passaged strain was recovered from spleen (Bp_mouse-adapted_). A gel-based comparative proteomics profiling approach was used, to map and identify differentially expressed proteins (fold-change ≥ 2; p-value ≤ 0.05) between the strains. A total of 730 and 685 spots were visualised in the Bp_*MARAN*_ and Bp_mouse-adapted_ strains, respectively. Of the 730 spots (Bp_*MARAN*_ as reference gel), 87 spots were differentially regulated (44 up- and 43 down-regulated). The identified proteins were classified as proteins related to metabolism, stress response, virulence, signal transduction, or adhesion. In comparison, it was found that those proteins related to adhesins, virulence factors and stress- response were up-regulated and could possibly explain the adaptation of the bacteria in the host. Investigating the differentially expressed proteins may provide better perspective of bacterial factors which aid survivability of *B. pseudomallei* in host.

## Introduction

Knowledge on the evolution and adaptation of intracellular bacteria within host during acute infection is still equivocal. The exact pathogenic mechanisms of disease caused by these bacteria are still not completely understood, especially the interaction between the host and the pathogen that results in an initial infections. The question on how intracellular bacteria adapt to the host environment and cause disease remains a challenge to researchers. Thus, we are interested to investigate the evolution during host-adaptation of intracellular bacteria *Burkholderia pseudomallei*, a free living saprophyte found in the soil and contaminated water. This bacterium is the causative agent of melioidosis and is commonly found in tropical and subtropical regions and endemic in Southeast Asian countries and northern Australia^1, 2^. The mode of transmission is through inhalation, ingestion, inoculation or direct entry of the organism into the blood stream via wounds or skin abrasions^3^. It is noteworthy to mention that to date, there is no vaccine has been invented to prevent the disease^4^.

*B. pseudomallei* is also known to produce exoenzymes, adhere, colonise, invade, replicate and survive intracellularly in the host cells^5, 6^. Due to the severe infection rate, aerosol infectivity and intrinsic resistant to a large number of antimicrobial agents, *B. pseudomallei* has been classified as a potential agent for bioterrorism (Tier 1 agent) by the U. S. Centers for Disease Control and Prevention and melioidosis has been indicated as an emerging infectious disease^7, 8^. Melioidosis manifest various clinical appearances ranging from chronic pneumonia, asymptomatic infection, multiple internal organ abscesses to septic shock^9–11^. Among the interesting features of *B. pseudomallei* infection is their ability to remain persistent with high rate of relapse incidence despite prolonged and appropriate antibiotic treatment^11^.

Investigations on the protein expression of the bacterium pre- and post-mouse-adaptation may provide better understanding of the role of the differentially expressed *B. pseudomallei* proteins that may be involved in pathogenesis of melioidosis. Therefore, *B. pseudomallei* isolated from soil would be an ideal choice of strain selection than a clinical strain as the strain has not adapted through a living system and compromised with immune responses. Although not a natural host for *B. pseudomallei*, mouse infection models play an important role in establishing the immunology and pathogenicity of *B. pseudomallei*.

Thus, experimental animal models that mimic human diseases are essential to provide information on etiopathogeny, immunity and therapy, as well as to improve our understanding on ways *B. pseudomallei* can induce a diverse range of diseases. Among various animal models available, mouse remains the most readily used animal model for studying *B. pseudomallei*-induced diseases and have known to play important roles in the elucidation of factors required for colonisation, distribution and persistence of infection^12^. Thus, in this study, it is of interest we analysed the *B. pseudomallei* protein factors potentially involved in the pathogen colonisation progression by using 2-DE and MALDI-TOF/TOF mass spectroscopy to perform a global comparative proteomic analysis of the *B. pseudomallei* obtained from soil and its derivate passaged through host immune system. This may shed light on a detailed understanding of the evolution and adaptation of *B. pseudomallei* within the host during infection. This will no doubt aid in identification of novel proteins related to the pathogenesis of disease that can be further utilized for clinical benefits in terms of diagnosis, protein markers or even vaccine development.

## Methods

### Bacterial strains, cell lines and culture conditions

The *B. pseudomallei MARAN* (Bp_*MARAN*_), isolated from soil at Lubuk Yu, Maran, Pahang, during a melioidosis outbreak was obtained from Institute of Medical Research (IMR), Kuala Lumpur, Malaysia. This strain was identified as *B. pseudomallei* using API 20NE (Biomerieux, France), Ashdown agar^13^ and an in-house PCR using specific primers^14^. The bacterial cultures of Bp_*MARAN*_ and *B. pseudomallei MARAN* mice-adapted strains (Bp_mouse-adapted_) were prepared using Luria-Bertani (LB) broth according to protocols earlier described^15^. The bacterial growth curve and viable counts were performed according to Mariappan *et al*.^16^ in three independent experiments. The human epithelial cell line, A549, cells were obtained from the American Type Culture Collection (Manassas, VA) and grown in Roswell Park Memorial Institute (RPMI) medium supplemented with 10% fetal calf serum (FCS). The cell line was grown under an atmosphere of 5% CO_2_.

### Determination of 50% lethality dosage (LD_50_)

For the virulence assessment, determination of LD_50_ was performed using five BALB/c mice (six-weeks to eight-weeks old) per group, which were intraperitoneally challenged with a 100 μl suspension of a diluted mid-logarithmic phase culture of Bp_*MARAN*_ and Bp_mouse-adapted_ corresponding to 10^4^ to 10^7^ CFU/ml. A group of five mice injected with sterile PBS were used as negative control. The mice were monitored daily (twice a day) and the number of dead mice in each group was determined up to 21 days post-infection. The LD_50_ values were calculated based on three independent experiments. The animal work was carried out in accordance with the approved guidelines by Association for Assessment and Accreditation of Laboratory Animal Care (AAALAC) International and the animal experimental protocols were approved by University of Malaya Institutional Animal Care and Use Committee [Animal Ethics: 2014-08-05/MMB/R/JSV]. Mice were euthanised according to pre-determined humane end point protocols supplied by the University Malaya IACUC at the end of the test duration.

### Mice infection of *B. pseudomallei*

Six six-weeks-old pathogen free male BALB/c mice were challenged via the intraperitoneal route with 10^5^ CFU/ml of Bp_*MARAN*_ culture resuspended in phosphate buffered saline (PBS). The mice were sacrificed on day-three and the spleen was harvested. The spleen was homogenised with sterilised PBS and the supernatant was inoculated onto an Ashdown agar. A single colony of the Bp_mouse-adapted_ strain was confirmed using API 20NE and an in-house PCR using specific primers. Spleens of the unchallenged mice were used as negative control.

### Adherence and invasion assays

The adherence and invasion assays were preformed according to Mariappan *et al*.^16, 17^ with slight modifications. The A549 cells were infected with Bp_*MARAN*_ and Bp_mouse-adapted_ strains grown to mid-logarithmic phase. The bacterial cultures were centrifuged at 8,000 X *g* for 5 mins and the resulting pellet was incubated at 37 °C in 1 ml of RPMI for 30 mins. The amount of bacterial inoculum was standardised to ~1 × 10^8^ cfu/ml. Confluent monolayers of A549 cells were infected with the bacterial inoculum at multiplicity of infection (MOI) of 1:10 and incubated for 2 hrs at 37 °C in 5% CO_2_ to allow bacterial invasion. The monolayers were then washed using 100 mM PBS (pH 7.0), after which 3 ml of RMPI containing a combination of kanamycin (1 mg/ml) and gentamicin (1 mg/ml) was added into each well and incubated for 2 hrs at 37 °C to kill extracellular bacteria. Following incubation, the monolayers cells were washed three times with PBS and in addition, the final volume of PBS used to wash the monolayers was collected and plated on a nutrient agar to determine the number of live extracellular bacteria. The monolayer cells were then lysed with 0.25% Triton X-100 prepared in PBS to quantitate the intracellular bacteria. Serially diluted lysate were plated on NA to determine the bacterial colony counts^18^. This experiment was performed in triplicates and the results were averaged. A non-invasive strain of *Escherichia coli* strain, was used as a negative control.

### Biofilm formation assay

Quantitative biofilm formation assay was performed using crystal violet staining method as described previously by Ramli *et al*.^19^. Briefly, 1 µl of a bacterial culture was added into 100 µl of LB broth in each well of a sterile 96-well plate and incubated at 37 °C for 18 hrs, after which, 1 µl of culture from each well was transferred into a new 96-well plate containing 100 µl of fresh LB broth and further incubated at 37 °C for 18 hrs. Following incubation, the supernatant was removed and the wells were stained with 150 µl of 1% crystal violet for 30 mins. The stains was removed by washing the wells twice with 175 µl sterile distilled water and DMSO (175 µl) was added to each well and absorbance was measured at 570 nm.

### Total bacterial protein extraction

The total bacterial proteins extraction was performed according to Al-Maleki, *et al*.^20^ with slight modifications in three independent experiments. Briefly, the Bp_*MARAN*_ and Bp_mouse-adapted_ strains grown as described earlier^15^ to mid-logarithmic phase were centrifuged at 4 °C, 4,500 X *g* for 30 mins. The resulting bacterial pellets were washed with cold PBS, and resuspended in 1 ml of lysis buffer^16^. Subsequently, the bacterial cells were sonicated on ice at 22% amplitude at 1 s pulse intervals for 3 mins using an Ultrasonic Homogenizer (Omni Ruptor 4000, USA). The lysate was then centrifuged at 14,000 X g for 5 mins at 4 °C and the supernatants were collected, aliquated and stored at −80 °C until use. The total protein concentrations were determined using the Bradford protein assay method^21^.

### Two-dimensional gel electrophoresis (2-DE) and image analysis

The two-dimensional gel electrophoresis was performed according to Vellasamy *et al*.^22^. Briefly, a total of 450 μg protein sample with the rehydration buffer (8 M urea, 2% CHAPS, 0.002% bromophenol blue) was applied onto the IPG strips (pH 3–10, 13 cm) (GE Healthcare, Uppsala, Sweden) and rehydrated under mineral oil for 18 hrs. The proteins on the IPG strips were focused using an IPGphor system (GE Healthcare, Uppsala, Sweden). The strips were then transferred onto 12% SDS-PAGE for the second dimension electrophoresis. The resulting gels were stained using Hot Coomassie blue staining method (GE Healthcare, Uppsala, Sweden). Three independent biological growth experiments were performed to increase reproducibility of the results. The gels were scanned with an Image Scanner and analysed using the Progenesis SameSpot (Nonlinear Dynamics, Durham, NC). The gels of Bp_*MARAN*_ and Bp_mouse-adapted_ strains were compared and protein spots demonstrating changes in protein expression with a fold change ≥ 2 (p-value ≤ 0.05; ANOVA) as the threshold values for differential expression were then identified.

### Protein identifications and MALDI-TOF MS/MS analysis

Selected protein spots of interest were excised and pooled from the stained 2-DE gels and digested with solution of sequencing-grade modified trypsin (Promega, Madison, USA). The peptides released from the gel plugs were then sent to the Australian Proteome Analysis Facility, Australia for further analysis using MALDI-TOF/TOF MS (4800 Proteomics Analyzer, AB Sciex). The subsequent search settings were used: carboxymidomethylation of cysteine was fixed modification and oxidation of methionine was selected as variable modification; maximum number of missed cleavages: 1; peptide tolerance (peptide tol): ±0.6; peptide charge: +1.

### Protein identification and *in silico* analysis

The mass spectrometry of the proteins was identified using the MASCOT search engine (Matrix Science, London, UK). All searches were performed against the non-redundant NCBI library (http://ncbi.nlm.nih.gov) database comprising annotated proteins of *B. pseudomallei* K96243 as reference. The identified proteins were assigned into functional classes of based on Cluster of Orthologous Groups (COG) (http://www.ncbi.nlm.nig.gov/COG/old/palox.cgi?fun=all) and Gene Ontology (http://omictools.com/gene-set-analysis) for the proteins functional categories. The bioinformatics database (www.cds.dtu.dk) was used to predict the mode of secretion (SignalP v3.0), cellular localisation (PSORT) and protein domains (TMHMM v2.0). The bacterial pathways of the significantly differentially expressed genes (student’s t-test) were analysed using the Kyoto Encyclopaedia of Genes and Genomes (KEGG) database (http://www.genome.jp/kegg/).

### Quantitative real-time PCR (qRT-PCR)

The qRT-PCR was performed utilizing CFX96 Touch Real-Time PCR Detection System (BioRad Laboratories, USA), to verify and quantify the expression of ompW, groL, EF-Tu, extracellular ligand binding protein, katG, Hsp, hypothetical protein BPSS1107and BPSL1958, with 50S and 30S ribosomal protein as reference housekeeping gene (Table S1). Briefly, 25 µl reactions were performed using the iScript™OneStep RT-PCR kit with SYBR green according to the manufacturer’s instruction (BioRad Laboratories, USA), primers at a final concentration of 1 µM and a data acquisition temperature of 56 °C. In order to control the variation in RNA concentration, cycle threshold (Ct) values were normalised to housekeeping genes that does not change with infection. The CFX real-time PCR software (Biorad, California, USA) was used to generate the quality control of the replicates, data extraction and initial analysis with a manual threshold of 0.6 and an auto baseline applied in order to obtain the threshold cycle (Ct) value for each measurement taken.

## Results

### Mice *Burkholderia pseudomallei* infection model

The growth profile of both strains were studied and revealed almost similar prolife in a nutrient rich medium under an aerobic condition (Supplementary Fig. S1). In this study, BALB/c mice were infected with Bp_*MARAN*_ and Bp_mouse-adapted_ strains via the intraperitoneal route. Based on the LD_50_ (Supplementary Fig. S2), the time to achieve 100% death from 10^7^ CFU/ml of Bp_*MARAN*_ was in two days; while with 10^6^ only one mouse survived on ﻿day﻿ 16. Approximately 60% of mice injected with 10^4^ CFU/ml survived until day 12. The LD_50_ of Bp_*MARAN*_ and Bp_mouse-adapted_ are 10^4^ and 10^4.6^ CFU/ml, respectively. It was found that upon infection, the mice infected with a dosage of >10^4^ CFU/ml of Bp_*MARAN*_ and Bp_mouse-adapted_ displayed symptoms of disease such as lethargy and had dishevelled fur before succumbed to infection. Two and three of the mice infected with Bp_*MARAN*_ and Bp_mouse-adapted_ survived up to 21 days, respectively. Overall, Bp_*MARAN*_ was found to be highly virulent than the Bp_mouse-adapted_ strain.

### Ability of Bp_*MARAN*_ and Bp_mouse-adapted_ strains to adhere and invade theA549 epithelial cells

We further investigated the ability of Bp_*MARAN*_ and Bp_mouse-adapted_ strains (mid-logarithmic phase) to adhere and invade into the A549 cells (Table 1). Generally, percentage of adherence increased in similar trend for both the strains corresponding with the time of infection. At 2 hrs and 4 hrs post-infection, there were no obvious differences observed in the number of Bp_*MARAN*_ and Bp_mouse-adapted_ adhered to the A549 cells (2.22 ± 0.96% and 16.67 ± 0%, respectively). The mean percentage of adherence of Bp_*MARAN*_ and Bp_mouse-adapted_ increased significantly from 6 hrs to 8 hrs with 2-fold and 1.5- fold changes, respectively. Overall, the percentage of adherence of Bp_mouse-adapted_ was noticeably higher than the Bp_*MARAN*_. In addition, the invasion profile was somehow similar to the adherence profile, where significant differences between these strains were observed at 6 hrs of post-infection. In general, the mean percentage of Bp_*MARAN*_ invasion was lower (0.2–0.3 fold) compared to the Bp_mouse-adapted_. Taken as a whole, the percentage of adherence and invasion were found to be strain-dependent.

**Table 1:** Mean percentage of *Burkholderia pseudomallei MARAN* and mouse-adapted adherence and invasion.

Time (Hours)	Adherence (%)		Invasion (%)	
	Bp_*MARAN*_	Bp_mouse-adapted_	Bp_*MARAN*_	Bp_mouse-adapted_
2	2.22 ± 0.96	2.22 ± 0.96	0.07 ± 0.05	0.10 ± 0.04
4	16.67 ± 0	16.67 ± 0	0.42 ± 0.28	0.47 ± 0.21
6	22.78 ± 1.83*	28.89 ± 1.92*	0.98 ± 0.09*	1.26 ± 0.11*
8	25.22 ± 2.89*	32.11 ± 1.25*	2.47 ± 0.87	3.50 ± 0.3

Foot note: *p-value ≤ 0.05.

### Aptitude of Bp_*MARAN*_ and Bp_mouse-adapted_ strains to produce of biofilm

**Figure 1:**
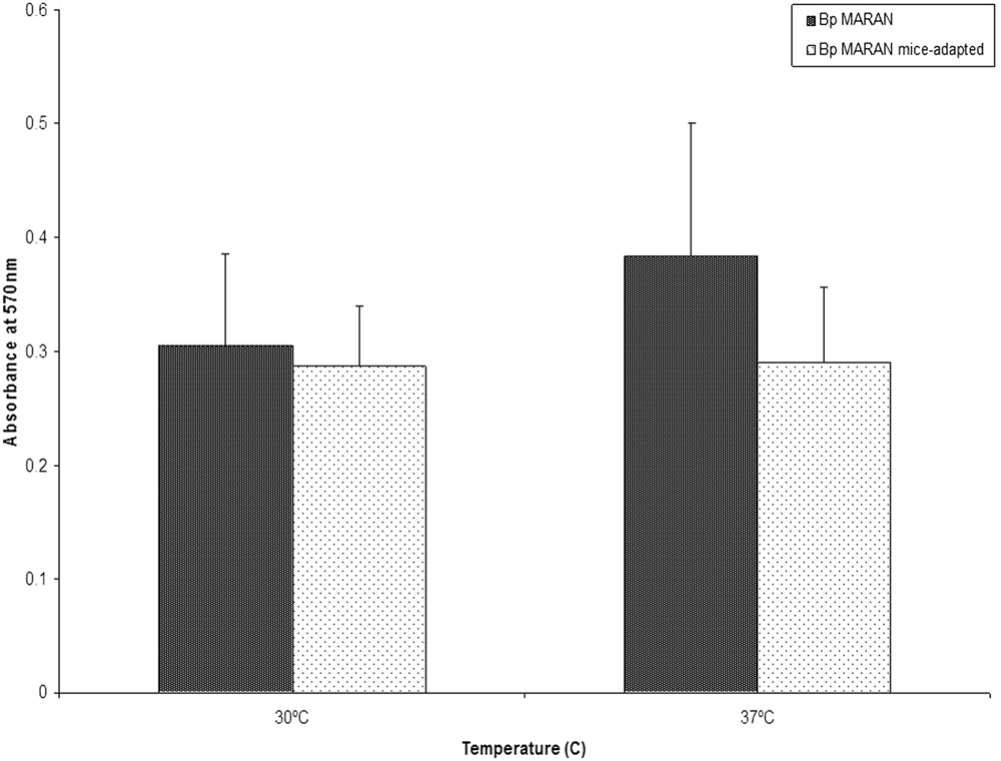
Biofilm formation of Bp_*MARAN*_ and Bp_mouse-adapted_ at 30 °C and 37 °C using crystal violet method. The experiments were conducted in three independent replicates (the error bars indicate the standard deviation and the significance has been indicated using*).

To investigate whether both Bp_*MARAN*_ and Bp_mouse-adapted_ strains are able to form biofilm at different temperatures, we determined the production the biofilm using the colorimetric method. In general, both the strains were found to produce biofilm at both 30 °C and 37 °C (Fig. 1). However, there were no significant differences observed in biofilm formation between Bp_*MARAN*_ and Bp_mouse-adapted_ strains at 30 °C and 37 °C. At 37 °C, the production of Bp_*MARAN*_ biofilm appeared to be increased (OD_570nm_ 0.384) as compared to the Bp_mouse-adapted_ (OD_570nm_ 0.291).

### Overview and comparative proteome analysis of Bp_*MARAN*_ and Bp_mouse-adapted_ strains

**Figure 2:**
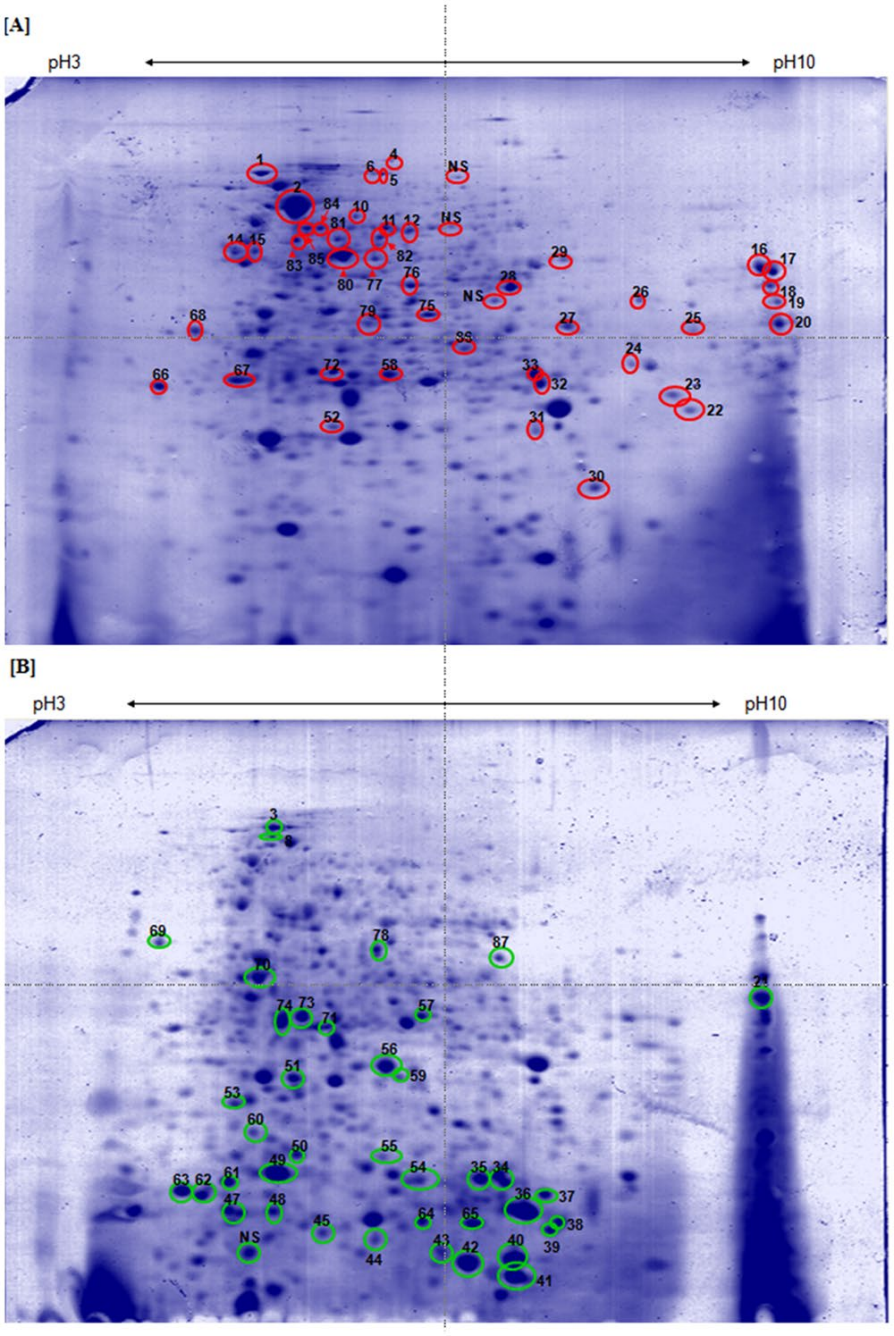
Analysis of Bp_*MARAN*_ and Bp_mouse-adapted_ whole bacterial protein by 2-DE. The whole bacteria of Bp_*MARAN*_ (panel A) and Bp_mouse-adapted_ (panel B) grown to mid-logarithmic phase in LB medium were prepared using sonication method and analysed using 2-DE. A total of 450 μg of whole bacteria was separated on an IPG strip pH 3–10 in the first dimension, followed by the separation on SDS-12% PAGE for the second-dimension separation. The separated proteins were detected by CBB G-250 staining. Marked spots indicate proteins that were identified (red: up-regulated; and green: down-regulated). NS refers to spots that were not possibly identified by MALDI-TOF.

In order to determine the protein expressions of these strains, we performed a comparative proteome analysis. Figure 2 shows the overview of whole bacterial Bp_*MARAN*_ (panel A) and Bp_mouse-adapted_ (panel B) 2-DE proteome profiles harvested from cultures grown at mid-logarithmic. The gel-based proteomics profiling approach was used to map and identify the differentially expressed proteins (fold change ≥ 2; p-value ≤ 0.05) between both the strains. A total of 730 and 685 protein spots were visualised from Bp_*MARAN*_ and Bp_mouse-adapted_, respectively. Approximately 594 spots were found to be present between the two strains.

However, of the 730 protein spots detected from the Bp_*MARAN*_ (reference gel), 87 protein spots were differentially regulated as compared to Bp_mouse-adapted_. Among these 87 protein spots, 44 spots were significantly up-regulated (fold change 2.0–12.2) and 43 were significantly down-regulated (fold change 2.0–6.9).

Of the 87 differentially regulated protein spots detected in the 2-DE profiles, only 83 (44 up-regulated and 39 down-regulated protein spots) were subsequently identified using MS and database search (Table 2). The remaining 4 protein spots were of low abundance and in insufficient quantities for the MALDI-TOF/TOF analysis. Overall, 44 up-regulated protein spots corresponding to 37 different proteins and 39 down-regulated protein spots corresponding to 25 proteins were identified. Following that, several proteins were found to be isoforms of the same proteins, *i.e*. DnaK, KatG, gltI, acetoacetyl-CoA reductase and hypothetical protein BPSS1924 (two isoforms); atpD and hypothetical protein BPSL1125 (three isoforms); Hsp (four isoforms); GroEL (six isoforms); EF-Tu (seven isoforms). It was noticeable that seven protein spots corresponding to five proteins [EF-Tu (n = 3), DnaK, Hsp, hypothetical protein BPSS1107 and hypothetical protein BPSS1158] were found to be absent from Bp_*MARAN*_, but present in Bp_mouse-adapted_. In contrast, 13 protein spots corresponding to 12 proteins [clpB, katG (n = 2), Alededh, PotF, PcaF, OmpW, Ppa, PixA, GroEL, MetX, ArgC and extracellular ligand binding protein] were found to be missing from Bp_mouse-adapted_, but present in Bp_*MARAN*_.

**Table 2:** Identification of *Burkholderia pseudomallei* soil isolate, Bp_*MARAN*_, up-regulated proteins in National Centre for Biotechnology (NCBI) non-redundant sequence using Mascot search engine data from MALDI-TOF mass spectrometry.

Functional category and Protein name^a^	Spot	Anova (p-value)	Locus tag^b^	Fold change	Sequence coverage(%)	Theo/exp MW	Theo/exp pI	SignalP^d^	TMHMM^f^	Subcellular location^c^
**Metabolism**										
***Energy production and conversion***										
F0F1 ATP synthase subunit alpha (atpA)	10	0.003	BPSL3398	2.0	20	55891/55781	5.48/5.48	−	−	cytoplamic
Betaine aldehyde dehydrogenase (alededh)	11*	6.15E-04	BPSL1550	3.2	30	50453/50353	5.67/5.67	−	−	cytoplamic
Isocitrate lyase (aceA)	12	0.042	BPSL2188	2.0	12	52238/47644	7.68/5.73	−	−	cytoplamic
Pyrophosphatase (ppa)	52*	0.026	BPSL1021	2.5	23	19149/19030	5.37/5.37	−	−	cytoplamic
Isocitrate dehydrogenase (idhI)	77	0.043	BPSL0896	2.0	24	46116/46015	5.76/5.63	−	−	unknown
F0F1 ATP synthase subunit beta (atpD)	83	0.013	BPSL3396	2.0	21	50417/50492	5.24/5.26	−	−	cytoplamic
F0F1 ATP synthase subunit beta (atpD)	84	0.018	BPSL3396	2.0	20	50147/50492	5.24/5.26	−	−	cytoplamic
Succinate dehydrogenase Iron-sulfur subunit (sdhB)	85	0.007	BPSS1717	2.0	21	26776/26633	6.00/6.00	−	−	cytoplamic membrane
***Amino acid transport and metabolism***										
Extracellular ligand binding protein (livK)	16*	0.004	BPSS0802	12.2	34	38875/39791	8.75/9.17	+	−	periplasmic
Putrescine-binding periplasmic protein (potF)	17*	0.017	BPSL1555	3.9	23	40171/40035	9.03/9.03	+	−	periplasmic
Periplasmic amino acid binding transport protein (livK)	18	0.038	BPSL3388	5.1	21	39974/39897	9.03/9.03	+	−	periplasmic
Argininosuccinate synthase (argG)	80	0.022	BPSL0298	2.0	24	49666/49566	5.375.37	−	−	cytoplamic
Arginine deiminase (arcA)	81	0.002	BPSL1743	2.1	32	46851/45921	5.40/5.57	−	−	cytoplamic
N-acetyl-gamma-glutamyl-Phosphate reductase (argC)	86*	0.005	BPSL3246	2.0	23	33676/33566	6.60/6.61	−	−	cytoplamic
***Carbohydrate and metabolism***										
Phosphopyruvate hydratase (eno)	14	0.049	BPSL2270	2.0	11	45656/45552	4.78/4.81	−	−	cytoplamic
Glyceraldehyde 3-phosphate dehydrogenase 1 (gapA)	28	0.03	BPSL2952	2.0	32	36082/35987	6.37/6.37	−	−	cytoplamic
***Coenzyme metabolism***										
S-adenosylmethionine synthetase (metK)	82*	2.31E-04	BPSL0212	3.7	16	42615/42510	5.13/5.13	−	−	cytoplamic
***Lipid metabolism***										
Beta-ketoadipyl CoA thiolase (paaJ)	29*	3.30E-04	BPSL3042	9.5	17	42285/42152	6.26/6.55	−	−	cytoplamic
Succinyl-CoA:3-ketoacid-coenzyme A transferase subunit A (scoA)	58	0.039	BPSL1955	2.0	20	25230/25087	5.56/5.55	−	−	cytoplamic
Succinyl-CoA:3-ketoacid-coenzyme A transferase subunit B (scoB)	67	0.048	BPSL1954	2.0	17	22273/22156	4.70/4.70	−	−	cytoplamic
***Inorganic ion transport and metabolism***										
Catalase-peroxidase (katG)	5*	0.002	BPSL2865	2.6	7	79339/81630	5.66/5.89	−	−	cytoplamic
Catalase-peroxidase (katG)	6*	0.029	BPSL2865	3.8	6	79339/81630	5.66/5.89	−	−	cytoplamic
Phosphate transport system, substrate-binding exported periplasmic protein (pstS)	20	0.031	BPSL1359	2.0	26	35762/36230	8.89/9.01	+	−	periplasmic
***Secondary metabolites biosynthesis, transport and catabolism***										
Non-ribosomally encoded peptide/polyketide synthase (phyH)	75	0.004	BPSS1183	2.0	24	39771/35275	7.73/5.77	−	−	cytoplamic
**Cellular Processes**										
***Cell wall/membrane***/***envelope biogenesis***										
Cell division protein (FtsZ)	15	0.008	BPSL3020	2.0	22	41574/41469	4.87/4.86	−	−	cytoplamic
Outer membrane protein (ompW)	31*	0.003	BPSL1552	3.1	17	22749/22632	8.60/8.60	+	−	outer membrane
***Post-translation modification and chaperones***										
Molecular chaperone (DnaK)	1	0.013	BPSL2827	2.9	15	69700/69571	4.66/4.93	−	−	cytoplamic
Chaperonin (GroEL)	2	0.009	BPSL2697	2.1	38	57080/56985	5.13/5.13	−	−	cytoplamic
ClpB heat-shock protein	4*	0.005	BPSL1484	2.2	9	95991/97147	5.66/5.66	−	−	cytoplamic
Chaperonin (GroEL)	27	0.021	BPSL2697	4.3	23	57486/56985	5.13/5.13	−	−	cytoplamic
Chaperonin (GroEL)	68	0.041	BPSL2697	2.2	19	57080/56985	5.13/5.13	−	−	cytoplamic
Chaperonin (GroEL)	72*	0.042	BPSL2697	2.0	18	57486/56985	5.13/5.13	−	−	cytoplamic
**Information Storage And Processing**										
***Translation, ribosomal structure and biogenesis***										
Ribosome recycling factor (frr)	22	0.039	BPSL2156	2.0	14	20886/20990	7.88/7.88	−	−	cytoplamic
50 S ribosomal protein L9 (rplI)	30	0.041	BPSL1461	2.1	50	16235/16114	6.85/6.85	−	−	cytoplamic
Elongation factor Tu (tuf)	79	0.037	BPSL3215	2.0	25	42964/42860	5.36/5.34	−	−	cytoplamic
***Transcription***										
DNA-directed RNA polymerase subunit alpha (rpoA)	76	0.048	BPSL3187	2.0	24	35663/35554	5.76/5.76	−	−	cytoplamic
**Poorly Characterised**										
***Function unknown***										
Hypothetical protein BPSS0837 (usp)	25	0.019	BPSS0837	2.8	16	29814/29832	8.04/8.05	−	−	unknown
Chitin-binding protein (cpbD)	26	0.048	BPSS0493	3.0	8	39260/39153	8.59/8.59	+	−	extracellular
BPSL0348 (pixA)	66*	0.042	BPSL0348	2.3	43	20170/27756	4.55/6.59	−	−	unknown
**Moonlighting proteins**										
***Amino acid transport and metabolism***/***Signal transduction mechanisms***										
Glutamate/aspartate periplasmic binding protein (gltI)	19	0.038	BPSL2924	2.9	33	32699/31300	9.17/9.17	+	−	periplasmic
Lysine-arginine-ornithine transport system, binding exported protein (argT)	24	0.044	BPSS0269	2.0	16	27833/28350	7.64/8.61	+	−	periplasmic
***Secondary metabolites biosynthesis, transport and catabolism***/***General function prediction***										
Acetyacetyl-CoA reductase (phbB)	32	0.036	BPSL1536	2.5	22	26722/26377	6.84/6.84	−	−	cytoplamic
Acetoacetyl-CoA reductase (phbB)	33	0.042	BPSS1916	2.1	26	26408/26298	6.60/6.30	−	−	cytoplamic
***Carbohydrate and metabolism***/***Cell wall***/***membrane***/***envelope biogenesis***										
Chain A, crystal structure of putative exported protein (wcaG)	23	0.039	−	2.0	27	25261/25582	8.41/8.12	−	−	unknown

^*^Missing proteins in Bpmouse-adapted.

^a^Functional category based on Clusters of Orthologous Groups (COG) of protein.

^b^Locus tag obtained from Burkholderia Database.

^c^Subcellular location predicted using the PSORT analysis.

^d^Presence of signal peptide predicted by SignalP server v. 3.0.

^e^Number of predicted transmembrane helices in protein using TMHMM server v. 2.0.

### **Identification of differently expressed Bp**_***MARAN***_**and Bp**_**mouse-adapted**_**proteins**

**Figure 3:**
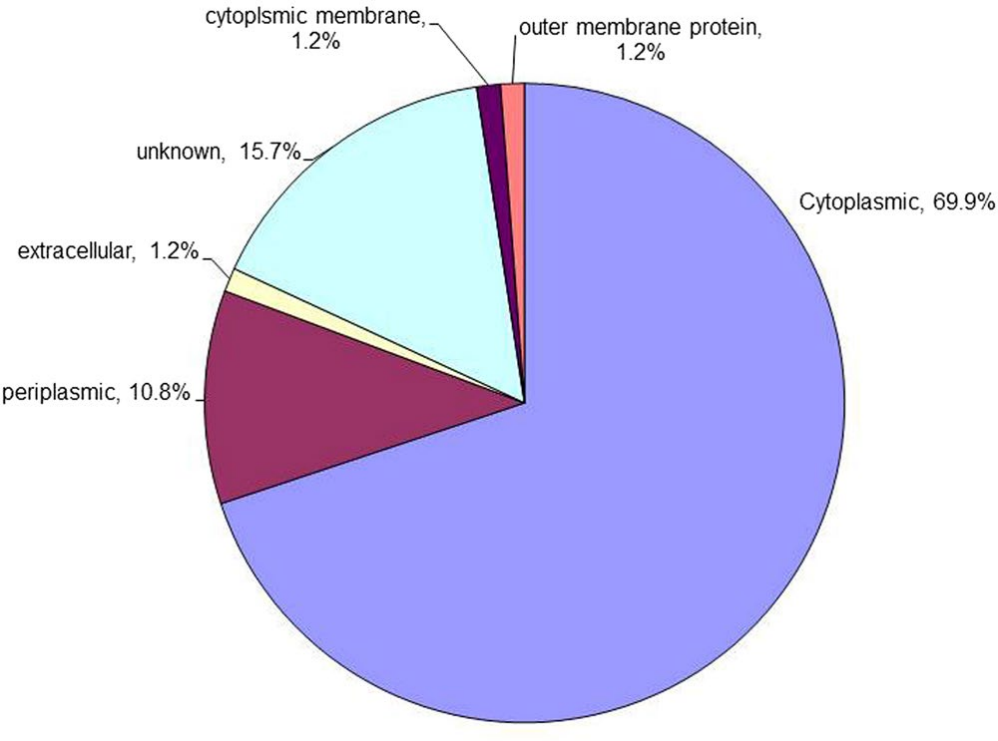
Subcellular localisation of differently expressed Bp_*MARAN*_ and Bp_mouse-adapted_ proteins. The subcellular localisation categories were predicted using PSORT.

Among the 83 differently regulated proteins identified, 58 proteins (69.9%) were identified to be located in the cytoplasmic region, nine proteins (10.8%) from the periplasmic compartment and 13 proteins (15.7%) had no known location. However, Cbp, SdhB, and OmpW were predicted to be located at extracellular, cytoplsmic membrane, and outer membrane, respectively (Fig. 3).

Of the five missing proteins from Bp_*MARAN*_, three proteins (EF-Tu, DnaK, and hypothetical protein BPSS1107) were located at cytoplasmic region and two proteins (Hsp and hypothetical protein BPSS1158) were predicted to be present at unknown location. However, out of the 12 proteins which were found to be missing from Bp_mouse-adapted_, eight proteins (ClpB, KatG, Alededh, PcaF, Ppa, GroEL, MetX and ArgC) were located at the cytoplasmic region, two proteins (PotF and extracellular ligand binding protein) at the periplasmic, OmpW were located at the outer membrane and PixA, is a protein with unknown location.

Further investigation using SignalP v3.0 (Table 3) predicted the presence of cleavage sites for bacterial signal peptidases, 10 commonly expressed proteins (i.e PotF, GltI, ArgT, Cbp, OmpW, DUF3315, periplasmic amino acid binding transport protein, extracellular ligand binding protein, phosphate transport system substrate-binding exported periplasmic protein, hypothetical protein BPSL1125) were predicted as secretory proteins of the classical Sec pathway. As expected, SignalP was predicted to present on the protein located in the outer membrane and the extracellular proteins. However, the TMHMM v2.0 algorithm predicted phosphate transport system substrate-binding exported periplasmic protein as integral membrane-associated proteins with only one helix (Table 3).

**Table 3:** Identification of *Burkholderia pseudomallei* soil isolate, Bp_*MARAN*_, down-regulated proteins in National Centre for Biotechnology (NCBI) non-redundant sequence using Mascot search engine data from MALDI-TOF mass spectrometry.

Functional category and Protein name^a^	Spot (p-value)	Anova tag^b^	Locus change	Fold	Sequence coverage (%)	Theo/exp MW	Theo/exp pI	SignalP^d^	TMHMM^f^	Subcellular location^c^
**Metabolism**										
***Energy production and conversion***										
Quinone oxidoreductase (qor)	87	0.012	BPSL1169	−2.1	13	34706/34598	6.97/6.25	−	−	cytoplamic
***Amino acid transport and metabolism***										
Acetylornithine transaminase (argD)	57	0.002	BPSL0926	−2.0	19	42851/42761	5.55/5.55	−	−	cytoplamic
***Lipid metabolism***										
Enoyl-CoA hydratase (paaG)	73	0.011	BPSL3043	−2.5	33	28383/28269	5.31/5.32	−	−	cytoplamic
***Inorganic ion transport and metabolism***										
Superoxide dismutase (sodB)	56	0.036	BPSL0880	−2.0	15	21159/21013	5.74/5.74	−	−	periplasmic
***Secondary metabolites biosynthesis, transport and catabolism***										
Catechol 1,2-dioxygenase (catA)	78	0.001	BPSS1892	−6.1	32	32947/32836	5.62/5.62	−	−	cytoplamic
**Cellular Processe**										
***Post-translation modification and chaperones***										
Trigger factor (tig)	8	0.037	BPSL1402	−2.0	28	49708/49637	5.00/5.00	−	−	cytoplamic
Chaperonin (GroES)	41	0.021	BPSL2698	−2.3	12	10484/10359	5.78/5.79	−	−	cytoplamic
Hydroperoxide reductase (AhpC)	48	0.004	BPSL2096	−2.4	30	20322/20174	5.05/5.05	−	−	cytoplamic
Heat shock protein 20 (Hsp)	49	8.92E-04	BPSS2288	−2.3	22	15716/15895	5.14/5.14	−	−	unknown
Chaperonin (GroEL)	53	0.043	BPSL2697	−3.0	16	57486/56985	5.13/5.13	−	−	cytoplamic
Molecular chaperone DnaK	54*	0.003	BPSL2827	−3.5	8	69659/69571	4.94/4.93	−	−	cytoplamic
Heat shock protein 20 (Hsp)	61	5.54E-05	BPSS2288	−4.8	22	15716/15895	5.14/5.14	+	+	unknown
Heat shock protein 20 (Hsp)	62*	0.004	BPSS2288	−6.9	22	15716/15895	5.14/5.14	−	−	unknown
Heat shock protein 20 (Hsp)	63	0.015	BPSS2288	−6.4	22	15716/15895	5.14/5.14	−	−	unknown
Heat shock protein 20 (Hsp)	65	6.33E-04	BPSS2288	−5.0	26	15716/15895	5.14/5.14	−	−	unknown
Chaperonin (GroEL)	70	0.003	BPSL2697	−2.3	19	57080/56985	5.13/5.13	−	−	cytoplamic
***Signal transduction mechanisms***										
dksA/traR C4-type zinc finger family protein (dksA)	50	0.025	BPSL0205	−2.4	56	15972/15879	5.11/5.24	−	−	cytoplamic
**Information Storage And Processing**										
***Translation, ribosomal structure and biogenesis***										
30S ribosomal protein S1 (rpsA)	3	0.049	BPSL2515	−2.0		62300/62177	4.82/5.08	−	−	cytoplamic
PTS system, EIIA component (yhbH)	34	0.018	BPSL0532	−3.0	26	13668/13534	6.49/6.49	−	−	cytoplamic
Elongation factor Tu (tuf)	35	0.02	BPSL3215	−2.1	12	42964/42860	5.36/5.34	−	−	cytoplamic
Elongation factor Tu (tuf)	37	0.047	BPSL3215	−2.2	10	42907/42860	5.41/5.34	−	−	cytoplamic
Elongation factor Tu (tuf)	44*	0.003	BPSL3215	−3.8	8	43597/42860	5.36/5.34	−	−	cytoplamic
Elongation factor Tu (tuf)	45*	0.001	BPSL3215	−3.6	8	43597/42860	5.36/5.34	−	−	cytoplamic
Elongation factor Tu (tuf)	60*	3.81E-04	BPSL3215	−5.2	7	43090/42860	5.36/5.34	−	−	cytoplamic
Elongation factor Tu (tuf)	64	0.005	BPSL3215	−4.2	8	4359742860	5.42/5.34	−	−	cytoplamic
Elongation factor Tu (tuf)	74	0.001	BPSL3215	−6.1	10	42953/42860	5.41/5.34	−	−	cytoplamic
***Transcription***										
Cold shock-like protein (csp)	42	0.005	BPSL0898	−4.8	14	7217/7090	6.54/6.54	−	−	cytoplamic
**Poorly characterised**										
***General function prediction***										
Hypothetical protein BPSS1924 (osmY)	51	0.012	BPSS1924	−2.3	18	20941/23037	5.68/5.31	−	−	cytoplamic
***Function unknown***										
Hypothetical protein BPSL1125 (DUF3315)	36	0.002	BPSL1125	−2.5	24	28032/13628	10.28/9.15	+	−	unknown
Hypothetical protein BPSL1125 (DUF3315)	38	0.041	BPSL1125	−2.3	6	28032/13628	10.28/9.15	+	−	unknown
Hypothetical protein BPSL1406	39	0.02	BPSL1406	−2.3	33	12347/12224	6.90/6.91	−	−	cytoplamic
Hypothetical protein BPSL1125 (DUF3315)	40	0.003	BPSL1125	−4.2	19	28032/13628	10.28/9.15	+	−	unknown
Phasin−like protein (phaP)	47	0.039	BPSL2298	−3.2	38	19861/19742	5.96/5.96	−	−	periplasmic
Hypothetical protein BPSS1107	55*	0.003	BPSS1107	−2.5	19	17045/16924	5.73/5.73	−	−	cytoplamic
Hypothetical protein BPSL1958	69*	0.043	BPSL1958	−2.8	3	36088/35995	4.38/4.38	−	−	unknown
Hypothetical protein BPSS1924 (osmY)	71	0.042	BPSS1924	−2.0	13	20941/23037	5.68/5.31	−	−	cytoplamic
**Moonlighting proteins**										
***Amino acid transport and metabolism***/***Signal transduction mechanisms***										
Glutamate/aspartate periplasmic binding protein (gltI)	21	0.014	BPSL2924	−5.6	22	32699/35200	9.17/9.17	+	−	periplasmic
***Secondary metabolites biosynthesis, transport and catabolism***/***General function prediction***										
Acetoacetyl-CoA reductase (phbB)	43	0.012	BPSS1916	−3.3	14	26408/26298	6.60/6.30	−	−	cytoplamic
***Signal transduction mechanisms***/***Transcription***										
Response regulator protein (regA)	59	0.007	BPSL0202	−2.3	41	19769/19800	5.82/5.83	−	−	cytoplamic

^*^Missing proteins in Bp*MARAN*.

^a^Functional category based on Clusters of Orthologous Groups (COG) of protein.

^b^Locus tag obtained from Burkholderia Database.

^c^Subcellular location predicted using the PSORT analysis.

^d^Presence of signal peptide predicted by SignalP server v. 3.0.

^e^Number of predicted transmembrane helices in protein using TMHMM server v. 2.0.

### The putative functions of the *differentially expressed proteins*

**Figure 4:**
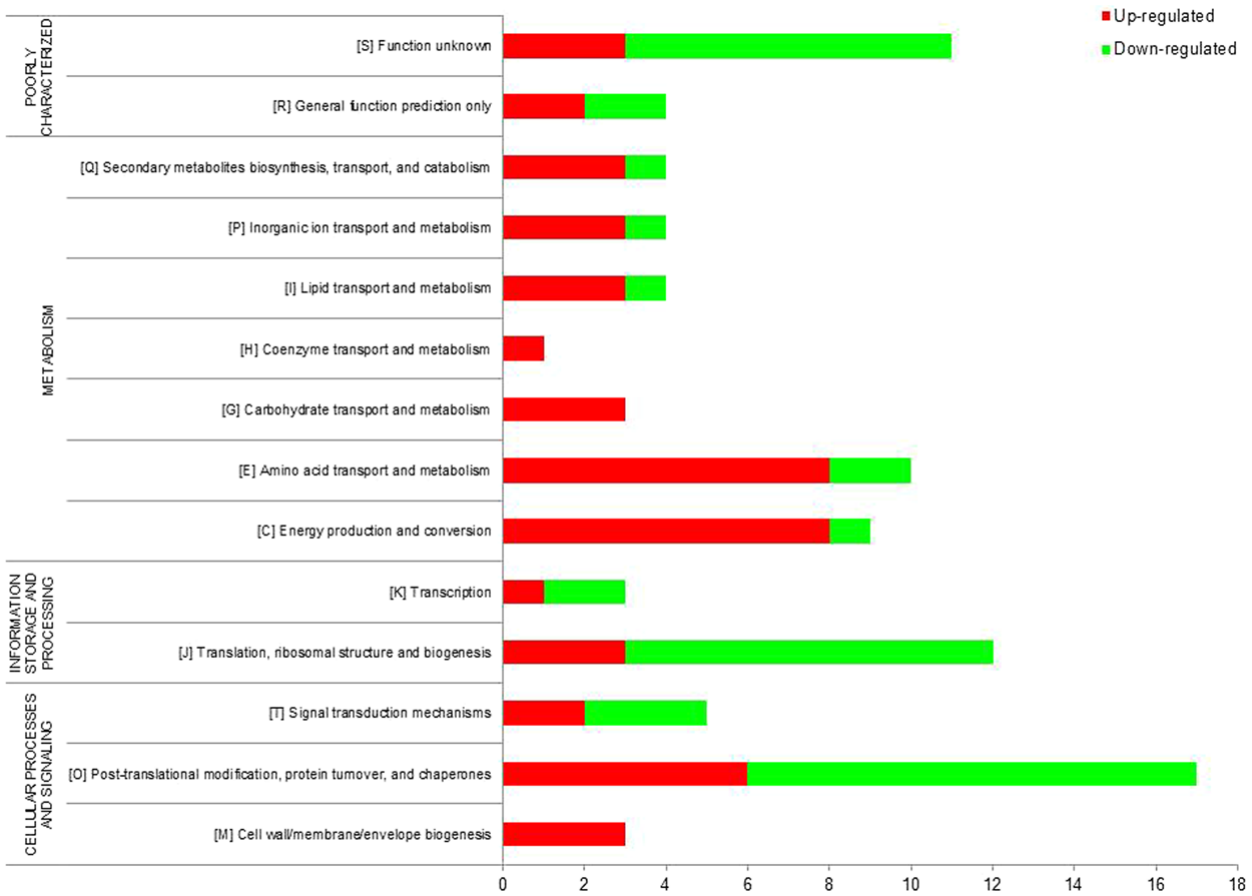
Prediction of the functional protein categories of differently expressed Bp_*MARAN*_ and Bp_mouse-adapted_ proteins. The identified proteins were assigned into functional classes of based on Cluster of Orthologous Groups (COG) and Gene Ontology (GO) for the proteins functional categories (metabolism, cellular processing and signal, information storage and processing and poorly characterised proteins). (red: up-regulated; green: down-regulated).

The identified proteins were analysed using COGs database to investigate the putative functions of the differently expressed proteins (Fig. 4). We predicted that majority of the up-regulated proteins (60.66%) were involved in metabolism [energy production (n = 8), amino acid transport (n = 13), carbohydrate transport (n = 4), coenzyme transport (n = 1), lipid transport (n = 5), inorganic ion transport (n = 3) and secondary metabolites biosynthesis/transport/and catabolism (n = 3)]. About 24.59% of the up-regulated proteins were involved in cellular processing and signal [cell wall/membrane/envelope biogenesis (n = 3), post-translational modification/protein turnover/chaperones (n = 6), and signal transduction mechanisms (n = 6)]. The remaining 6.56% and 8.20% were corresponding to information storage and processing and poorly characterised proteins, respectively.

In contrast, majority of the down-regulated proteins (38.30%) were involved in cellular processing and signal [cell wall/membrane/envelope biogenesis (n = 1), post-translational modification/protein turnover/chaperones (n = 11), and signal transduction mechanisms (n = 6)] and 23.40% were involved in information storage and processing [translation/ribosomal structure/and biogenesis (n = 9), and transcription (n = 2)]. Proteins that are poorly characterized were 19.15% (n = 9) and those involved in metabolism were only 19.15% (n = 9). Protein moonlighting (playing multiple unrelated functions) was also observed from several of the differentially expressed proteins; namely lysine-arginine-ornithine transport system, binding exported protein and glutamate/aspartate periplasmic binding protein (signal transduction mechanisms and amino acid transport and metabolism), chain A crystal structure of putative exported protein (cell wall/membrane/envelope biogenesis and carbohydrate transport and metabolism) and response regulator protein (signal transduction mechanisms and transcription). Acetoacetyl-CoA reductase and acetyacetyl-CoA reductase were also protein that moonlights having dual protein functions as secondary metabolites biosynthesis transport and catabolism, and general function prediction. Overall, there was a vast different, in term of the putative functional groups among the proteins that were differentially regulated between Bp_*MARAN*_ and Bp_mouse-adapted_.

### Pathways analysis of the differentially expressed proteins

To further analyse the putative functional roles of those differently expressed proteins, we included the KEGG pathway analysis (Table 4). Majority of the up-regulated pathways were found to be involved in metabolic functions, including microbial metabolism in diverse environments, glyoxylate and dicarboxylate metabolism, oxidative phosphorylation (energy metabolism) and butanoate metabolism (carbohydrate metabolism). Apart from that, several other pathways were also up-regulated, namely, ABC transporters (membrane transport - environmental information processing) and RNA degradation (folding, sorting and degradation - genetic information processing).

**Table 4:** Identification of the significant pathways (p-value < 0.05) of differentially expressed proteins using KEGG pathway analysis.

Condition	KEGG pathway	Number of gene (s)
***Up-regulated***	Metabolic pathways	14
	Microbial metabolism in diverse environments	7
	Oxidative phosphorylation	4
	Butanoate metabolism	4
	ABC transporters	5
	Biosynthesis of secondary metabolites	9
	Carbon metabolism	5
	RNA degradation	3
	Glyoxylate and dicarboxylate metabolism	2
***Down-regulated***	RNA degradation	3
	Arginine and proline metabolism	2
	2-Oxocarboxylic acid metabolism	2
	Two-component system	3
	Biosynthesis of amino acids	2
	Lysine biosynthesis	2

In contrast, several amino acid metabolism pathways were down-regulated, namely, 2-oxocarboxylic acid metabolism, biosynthesis of amino acids, lysine biosynthesis, arginine and proline metabolism. Apart from that, two-component system, which is involved in signal transduction (environmental information processing), RNA degradation (folding, sorting and degradation - genetic information processing) were also found to be down-regulated.

### Validation of the microarray data

**Figure 5:**
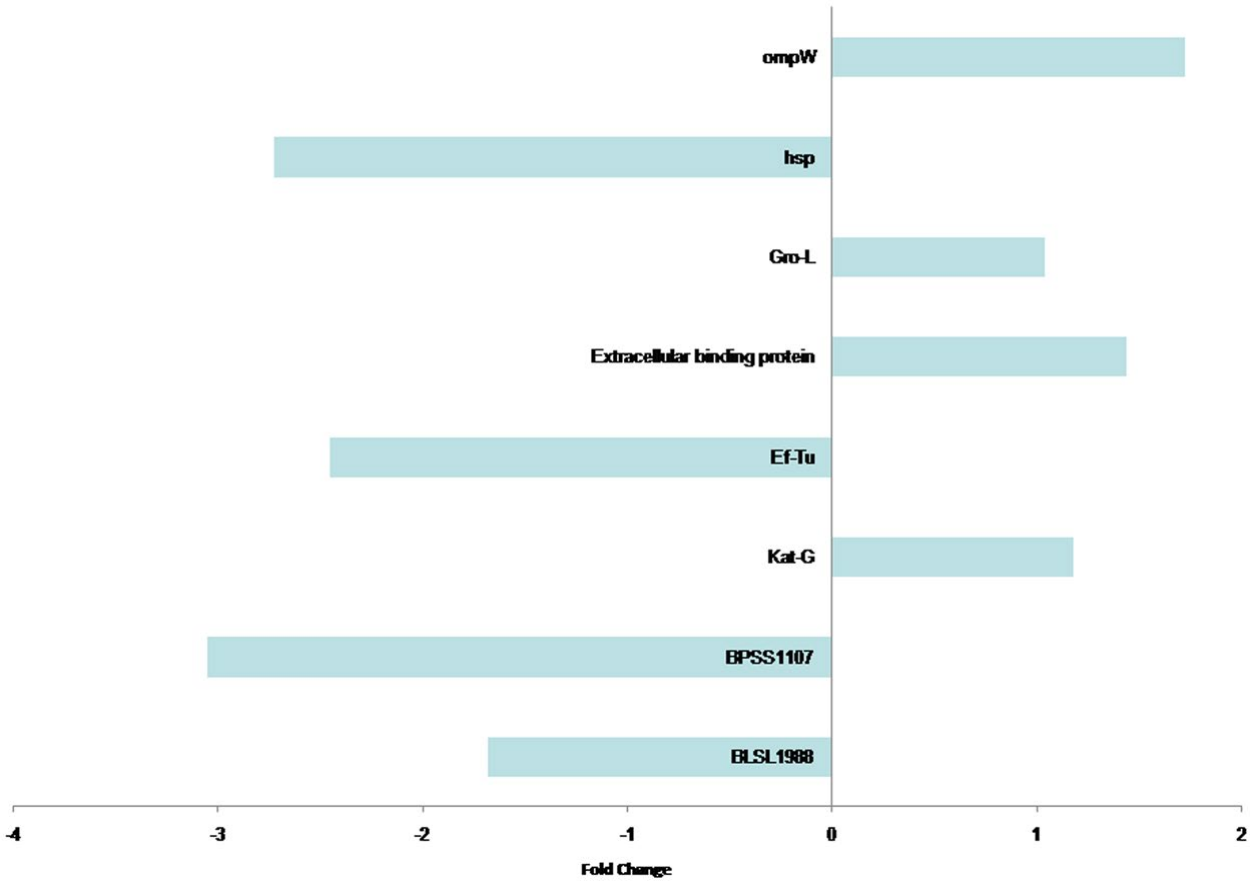
Quantitative real-time PCR (qRT-PCR) gene expression validation of those proteins regulated differently; namely ompW, groL, EF-Tu, extracellular ligand binding protein, katG, Hsp, hypothetical protein BPSS1107, BPSL1958 and BPSL0348 genes. Differences were observed in the fold-change values whereby the fold-change detected in qRT-PCR assay was higher than the protein expression.

The qRT-PCR assay was used for validation of ompW, groL, EF-Tu, extracellular ligand binding protein, katG, Hsp, hypothetical protein BPSS1107, BPSL1958 and BPSL0348 genes, which were differentially expressed in the proteomics analysis. The data obtained, together with those determined by the proteomic experiments are shown in Fig. 5. The gene expressions obtained using the two techniques were comparable. However, differences were observed in the fold-change values whereby the fold-change detected in qRT-PCR assay was higher than the protein expression.

## Discussion

Our study has demonstrated differential protein expression of *B. pseudomallei* isolated from soil and the same isolate subsequently passaged and harvested from the mice tested in the same laboratory growth condition. Comprehensive knowledge of the proteins expressed by *B. pseudomallei* during *in vivo* growth in the host is essential to understand the pathogenesis of infectious diseases and symbiotic process. During the infection process, *B. pseudomallei* are tackled with various stress factors including, nutrient starvation, antibiotics, heat/cold shock responses, and is presumably exposed to a variety of anti-bacterial factors such as complement, antibody, and phagocytes (which if activated, can obstruct progress of the bacteria to invade and infect the host).

Being a versatile intracellular bacteria, *B. pseudomallei*, would deliver its virulence factors into the host cells, depending on the close contact between the bacteria and the host^23^. On the other hand, the host is predisposed to exposure to the bacteria and counter-attack by the releasing immune responses^24^. Ultimately, this phenomenon alters the *B. pseudomallei* proteins expression to enable survival and persistence in the host under any extreme conditions. Thus, in order to identify the proteins associated with *B. pseudomallei* during colonisation in mice, we used 2-dimensional gel electrophoresis (2-DE) to analyse the whole bacterial proteins extracted from *B. pseudomallei* isolated from soil and the mouse-passaged homolog, which mimics an *in vivo* condition during infection. In addition, we also characterised different virulence factors of these strains including the ability to adhere, invade and capacity to produce biofilm.

Many studies have demonstrated the ability of clinical and environmental isolates of *B. pseudomallei* to adhere and invade into the cultured epithelial cells *in vitro* ^25–27^. Our finding suggested that the adherence and invasive abilities of mice adapted *B. pseudomallei* isolate was clearly different than its counterpart *B. pseudomallei* soil isolate, although the growth of both strains was increasing in a similar pattern. The percentage of adherence and invasion of Bp_mouse-adapted_ was noticeably higher than the Bp_*MARAN*_ and these differences may be attributed to the alteration in the adhesions proteins upon exposure to the host immune system. Collectively, it was evident that the Bp_mouse-adapted_ was more virulent and possess higher pathogenic potential compared to its counterpart the Bp_*MARAN*_. Similar findings have also been reported in *Helicobacter pylori* by Zhang *et al*.^28^, whereby the mice-adapted strain demonstrated higher ability to adhere and invade into host cells compared to the counterpart strains. However, both the strains were found to produce biofilm at both 30 °C (soil temperate) and 37 °C (host temperature). Significant differences were observed in biofilm formation at 37 °C whereby, lower biofilm in the Bp_mouse-adapted_ strain as compared with the soil strain (Bp_*MARAN*_). This could be due to stress caused by adaptation and compromise with the host immune responses.

To further investigate the altered proteins, we therefore performed 2-DE of Bp_*MARAN*_ and Bp_mouse-adapted_ whole bacterial proteins which were collected during the mid-logarithmic phase, when the bacterial cells are actively dividing. Interestingly, there was a slight difference in the number of proteins spots detected between both the strains. The number of protein spots identified in Bp_mouse-adapted_ was comparatively lower than the protein spots identified in its homolog counterpart strain. Several unique proteins were also found to be present in both the strains. This clearly demonstrates that the differences may be attributed to the variations of the bacterial protein modulations upon host-adaptation. In addition, several proteins involved in adherence and invasion were found to be up-regulated in the Bp_mouse-adapted_. Taken together these findings may indicate the contribution of the differentially expressed proteins to the ability of the strains to adhere and invade into host cells.

In our present study, the 2-DE protein profiles revealed a total of 87 protein spots that were differentially expressed between the *B. pseudomallei* Bp_*MARAN*_ and Bp_mouse-adapted_. Of that, 44 spots were found to be up-regulated and 43 were down-regulated. In general, majority of the proteins altered following direct interaction with the hosts were involved in metabolic functions. This could be due to the ability of *B. pseudomallei* to adjust the metabolic pathways in order to exploit nutrients from the host during the infection cycle and intracellular survival lifestyle^29, 30^. Several transportation and conversion of energy production, carbohydrate, lipid, coenzymes, inorganic ions and secondary metabolites systems may be vital for the survival of *B. pseudomallei*. Additionally, Stark *et al*.^31^ also found that those proteins identified in uptake of amino acids transporters were also seems to be essential during infection process of *H. pylori*. Thus, these may suggest that not only carbohydrate, but also amino acids could be obtainable as carbon source for energy consumption at certain point of infection as primary source of energy is scarce when *B. pseudomallei* undergoes metabolic stress^32^. Our results also revealed that the Bp_mouse-adapted_ had a restricted glycolysis/gluconeogenesis and increased in Bp_*MARAN*_. Similarly, a comparative proteomic study between *Brucella suis* (grown in rich media) and post-infected in J774 macrophages by Al Dahouk and co-workers^33^ demonstrated 44 differently expressed proteins involved in primary metabolism which is strictly needed for survival. Reduction of the metabolic pathways in Bp_mouse-adapted_ suggests that there may be limited sugar supply during the infection process. This in turn may aid in the persistence of the pathogen since by reducing or limiting the metabolic functions, it may be able to avoid recognition of the host immune system. In addition, the induction of proteins involved in oxidative phosphorylation suggested the use of alternative pathways for energy production^34^.

Although metabolic proteins are known to play major functions in energy production for survival, it has also been suggested that they play a role in virulence^35^. In this study, we also found that several proteins involved in virulence and persistence were significantly altered between the Bp_*MARAN*_ and Bp_mouse-adapted_. Expression of virulence proteins are usually controlled by signalling pathways and regulatory mechanism which is typically similar to those proteins that are indistinct to pathogenesis. It is often based on the reversible phosphorylation proteins, particularly the two-component regulatory system (consist of membrane-bound histidine kinase and response regulator) and phosphoenolpyruvate phosphotransfer system (PTS)^36^. In our study, the two-component system which facilitates *B. pseudomallei* to sense, respond, and adapt to changes in their environment or in their intracellular state were found to be up-regulated in Bp_mouse-adapted_. Activation of the two-component response regulator may induce changes in the cellular physiology, by regulating expression of different genes^37^, and thus, enabling *B. pseudomallei* to sense and respond to stimuli by inducing changes in transcription and virulence, particularly in biofilm formation. The inactivation of the transposition may eventually lead to defects in attachment, invasion and survival^38^. Additionally, Tuanyok *et al*.^32^ have identified differentially expressed potential virulence genes involved in the putative two-component regulatory system, and have also provided a better understanding of *B. pseudomallei* to generate and maintain cellular energy *in vivo* in response to the host environment during acute melioidosis using hamster model. It was also suggested that the PTS supply the bacteria with integrated system that ensure optimal utilisation of carbohydrate in complex environments, a feature that is particularly important in host-pathogen interaction^36^.

Apart from that, we also observed several proteins involved in virulence and/or persistence which were either absent or present in both Bp_*MARAN*_ and Bp_mouse-adapted_, particularly catalase/peroxidase (KatA). Lefebre *et al*.^39^ conclude that the catalase/peroxidase is has a novel function by contributing to maintain the normal activity of the tricarboxylic acid (TCA) cycle, and plays a global role in cellular protection against oxidative stress^40^. Modulation of outer membrane protein and several periplasmic transpoters were also observed, which may regulate homeostasis and structure of the *B. pseudomallei* envelope. The observations underline the significance of crucial pathogenesis of *B. pseudomallei* in terms of the interplay between bacterial counter-host immune mechanisms.

In summary, our results imply that both the host and the *B. pseudomallei* factors are involved in the development of the pathogenic outcome of infection. The identified proteins suggest differences in the pathogenic potentials of *B. pseudomallei* to infect and cause disease in man. Thus, this *in vivo* study provided a comprehensive description of the proteomic differences between the soil and mice-passaged *B. pseudomallei*, which is particularly imperative, since they revealed information that could not have been predicted using classic *in vitro* growth conditions.

Based on our analysis, the proteins that were differentially expressed between the *B. pseudomallei* soil strain and its mouse adaptive variant contributed to host colonisation and adaptation by modulating stress response, metabolism, virulence, and colonisation-associated proteins. The data on the role of these proteins in colonisation may highlight the importance of these proteins in the establishment of infection, provide information for the development of novel vaccine candidates, and indicate intervention modalities for the prevention or treatment of *B. pseudomallei* infections.

## Electronic supplementary material

Supplementary Data
