# Supplementary material for: Host-Adaptation of Burkholderia pseudomallei Alters Metabolism and Virulence: a Global Proteome Analysis
Source: Sci Rep. 2017 Aug 21;7:9015. doi: 10.1038/s41598-017-09373-0 (PMC5567125; doi:10.1038/s41598-017-09373-0)
Supplement: Supplementary file 1 — Supplementary Data [file 41598_2017_9373_MOESM1_ESM.pdf]

Host-Adaptation of *Burkholderia pseudomallei* Alters Metabolism and Virulence: a Global Proteome Analysis

Vanitha Mariappan[1], Kumutha Malar Vellasamy[1], Jamuna Vadivelu[1*]

[1]Department of Medical Microbiology, Tropical Infectious Diseases Research and Education Centre (TIDREC), Faculty of Medicine, University of Malaya, 50603 Kuala Lumpur, Malaysia

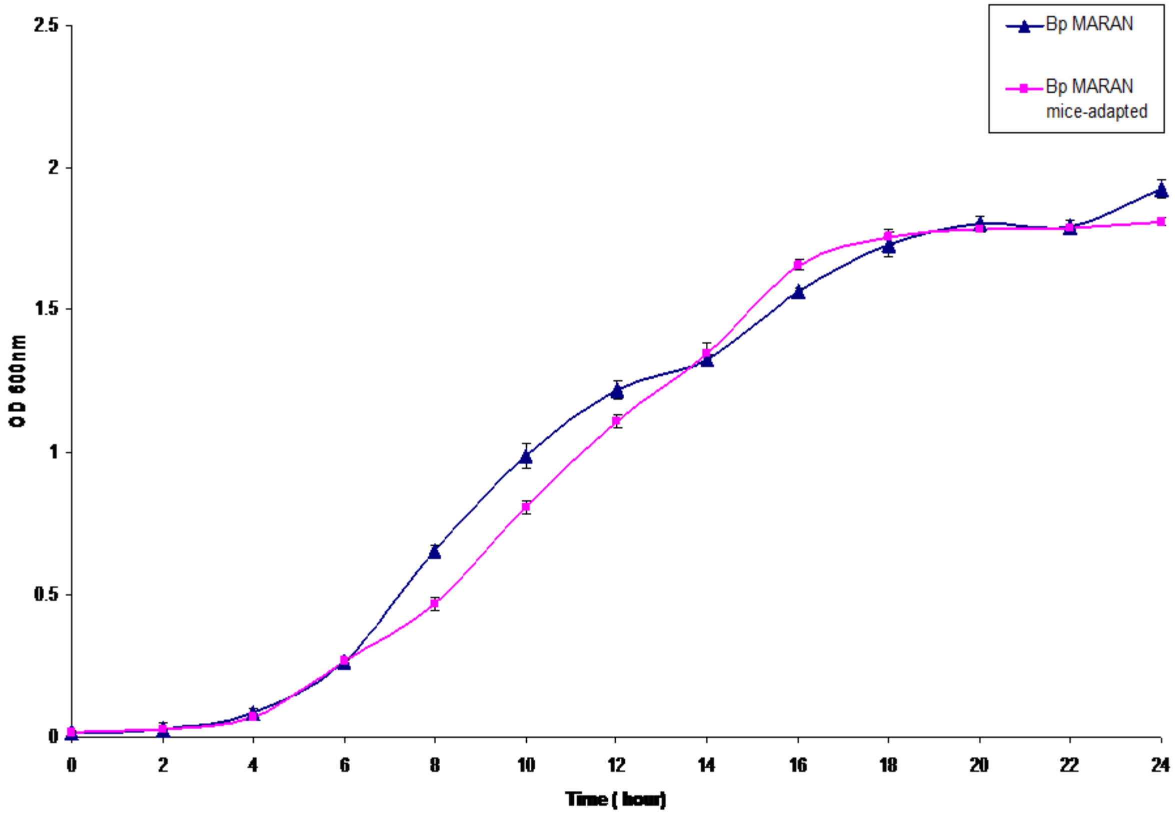

Supplementary Figure 1. Growth curve of Bp_{MARAN} and $Bp_{mouse\text{-}adapted}$. The culture was inoculated in LB medium and grown at 37°C from 0 h to 24 h. It was harvested at every 2 h intervals and the bacterial density was determined at OD_{600nm}. The experiment was conducted in three independent replicates (the error bars indicate the standard deviation).

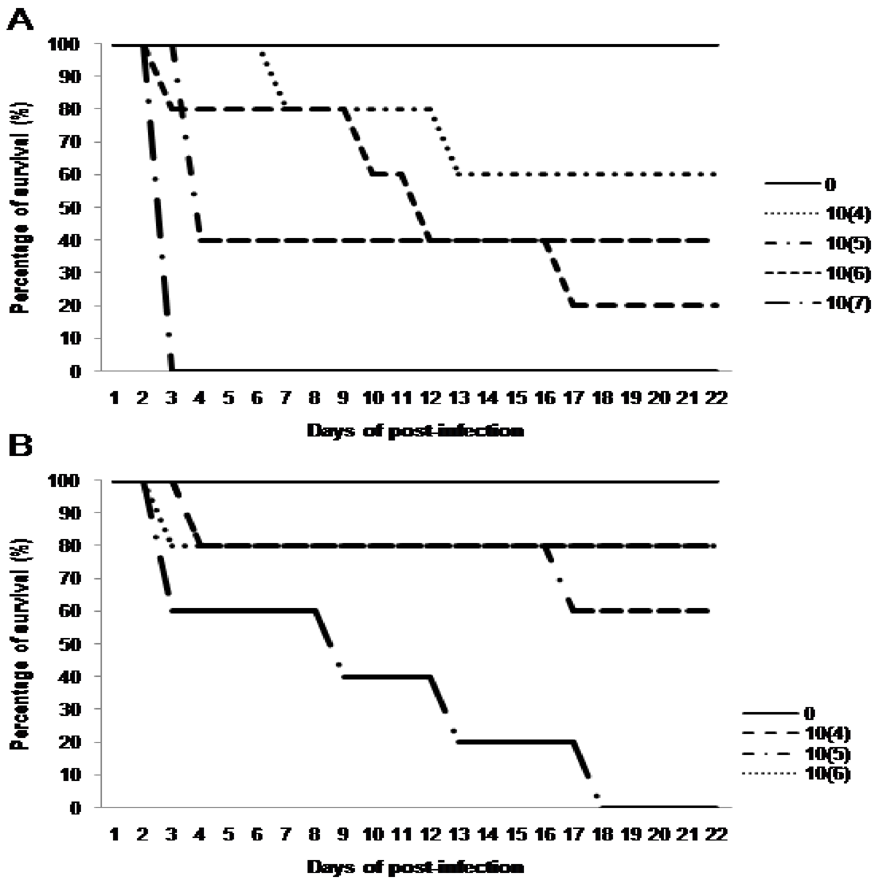

Supplementary Figure 2. LD_{50} of (A) Bp_{MARAN} and (B) $Bp_{mouse\text{-}adapted}$.

Table S1: List of primers used for gene expression

**Gene**	**Primer ((5'-3')**
catalase-peroxidase	F aagcgaagtgcccgttccat<br>R gtagttgaaatccttgccca
hsp 20	F gaagatcgacgtgaccgaaa<br>R gccttccttcagttccttgt
hypothetical protein BPSS1107	F gacaagcagaccggcgtgat<br>R caggctgatctgttcgtagtcg
hypothetical protein BPSL1958	F cgctcgcgacatcgaaccag<br>R ccgacaacccatgccacgtt
chaperonin GroEL	F cagttcgaccgcggctacctgtc<br>R aggtcgcggatgttcgacacct
elongation factor Tu	F gggcgtggaaatgttccgcaagc<br>R acccggcttcgccagaacctg
extracellular ligand binding protein	F acgaactacaccggcgtgat<br>R ttacatcgtcacctgatcgagg
outer membrane protein	F aatacgttgtcggcgctgaa<br>R gcatggttgaagtggtactgc
